# The feasibility of debranching aortic arch and visceral arteries with sutureless telescoping anastomoses during open aortic aneurysm repair

**DOI:** 10.1016/j.jvscit.2023.101159

**Published:** 2023-03-17

**Authors:** Elan A. Sherazee, Amir A. Sarkeshik, Matthew Vuoncino, Timothy M. Guenther, Victor M. Rodriguez

**Affiliations:** a Department of Surgery, University of California Davis, Sacramento, CA; b Division of Cardiac Surgery, Department of Surgery, University of California Davis, Sacramento, CA; c Division of Vascular Surgery, Department of Surgery, University of California Davis, Sacramento, CA

**Keywords:** Anastomosis, Ascending aortic aneurysm, Blood vessel prosthesis implantation, Surgical, Thoracoabdominal aortic aneurysm

## Abstract

**Background:**

Open repair of aortic aneurysms frequently requires reimplantation of major aortic vessels. Traditional techniques can be time consuming, require meticulous hemostasis, and risk aneurysmal patch degeneration, which can require a challenging reoperation. We describe our experience using a stent graft to create a sutureless anastomosis that obviates these drawbacks.

**Methods:**

Between April 2018 and March 2021, all consecutive adult patients who underwent open repair of the aorta with at least one supra-aortic trunk or visceral vessel reimplanted using the sutureless anastomotic technique were included. Anastomoses were constructed by bridging a branch graft and the target artery with a Viabahn self-expanding stent (W.L. Gore & Associates, Flagstaff, AZ). Clinical information and perioperative outcomes for the patients were collected and analyzed.

**Results:**

Among 26 patients, 50 individual aortic vessels were debranched using sutureless self-expanding stent anastomoses, including 42 visceral vessels and 8 supra-aortic trunk vessels. Technical success was 100%. The median time to complete the anastomosis was 3 minutes, 12 seconds (range, 2-6 minutes). Perioperative mortality was 15% (n = 4). No stent-related complications, such as occlusion, bleeding, stroke, renal failure requiring hemodialysis, bowel ischemia, or the need for anastomotic reintervention, occurred. Follow-up imaging at 1 year revealed a 100% patency rate and no anastomotic stenosis, misalignment, or kinking.

**Conclusions:**

The sutureless anastomosis technique to debranch the aorta during open aortic aneurysm repair is technically feasible and reliably hemostatic and does not require early reintervention. The operative outcomes have been acceptable, and the short-term follow-up imaging findings demonstrated excellent patency without anastomotic kinking. In select cases, sutureless anastomoses are a possible alternative to traditional sutured anastomoses during aortic debranching. Further research is needed to compare the operative times and long-term patency of sutureless anastomosis to those of traditional sutured techniques.

Reimplantation of visceral arteries and the supra-aortic trunks (SATs) during open aortic aneurysm repair is often accomplished by the Crawford inclusion technique, whereby the vessels are reimplanted as a large Carrel patch, directly anastomosed en bloc to the synthetic aortic graft.^1^^,^^2^ However, this technique leaves a considerable amount of native, diseased aorta that, not uncommonly, undergoes aneurysmal degeneration and could necessitate a challenging and morbid reoperation.^3^^,^^4^ Alternatively, to avoid patch aneurysms, individual branched grafts can be sewn to each target vessel. This branch graft approach, however, requires a greater number of sutured anastomoses that take longer to complete than a single patch, prolonging flow interruption and end organ ischemia. The use of multiple branched grafts can also increase the risk of anastomotic kinking during viscera derotation.^5^ An alternative approach to debranching the aorta that addresses these limitations is sutureless anastomosis.^6^ The concept of sutureless anastomosis relies on deploying a stent graft or fabric tube supported by a metal wire scaffold to quickly create a reliably hemostatic anastomosis and overcome technically challenging anatomy.^7^ In the present report, we describe our institution's experience debranching the aorta with self-expanding stent grafts to create sutureless anastomoses during open repair of thoracoabdominal aortic aneurysms (TAAAs) and aortic arch aneurysms.

## Methods

### Overview

The institutional review board approved the present study and access to the patients’ electronic health records to collect the minimum necessary protected health information for the study participants (approval no. 1681895-1; December 18, 2020). The institutional review board waived the requirement for patient informed consent for participation in the present study.

Retrospective data were collected on 26 consecutive patients who underwent open repair of the aorta with sutureless debranching between April 2018 and March 2021. All surgeries were performed by a single surgeon (V.M.R.) at an academic institution. The inclusion criteria were elective or urgent/emergent treatment of an aortic aneurysm and/or dissection by open repair and at least one sutureless anastomosis of a visceral artery (eg, celiac artery, superior mesenteric artery [SMA], left renal artery, right renal artery) or SAT (eg, innominate artery, left carotid artery, left subclavian artery). All the patients had provided written informed consent for aortic repair after provision of pertinent information, including the risks, benefits, and alternatives. In addition, they were informed of the possibility of using stent grafts to create certain anastomoses instead of suture. The demographics, comorbidities, and aortic pathologies of the patients included for analysis are presented in Table I.

**Table I:** Demographic patient characteristics

Variable	Value
Patients	26 (100)
Male gender	17 (65.4)
Age, years	62.5 (32-79)
Etiology	
Atherosclerotic/degenerative	12
Dissection	10
Connective tissue disorder	4
Previous cardiac intervention	14 (53.8)
Comorbidity	
Hypertension	19 (73.1)
Diabetes mellitus	2 (7.7)
Dyslipidemia	7 (26.9)
COPD	6 (23)
Cerebrovascular disease	5 (19.2)
Marfan syndrome	4 (15.4)
Chronic kidney disease	5 (19.2)
TAAA classification	18
I	2 (11.1)
II	6 (33.3)
III	3 (16.6)
IV	6 (33.3)
V	1 (5.5)
Arch aneurysm	8
Urgent/emergent	12 (46.2)

*COPD,* Chronic obstructive pulmonary disease; *TAAA,* thoracoabdominal aortic aneurysm.

Data presented as number (%), median (range), or number.

### Statistical analysis

Descriptive data analysis was performed using STATA, version 15 (StataCorp, College Station, TX). Categorical data are described using absolute numbers and percent prevalence. Continuous variables are presented as the median and range.

### Indications

The sutureless anastomosis is our default choice to debranch the visceral arteries and SATs. However, ultimately, the operating surgeon had discretion regarding whether to perform a sutureless or sutured anastomosis.

### Contraindications

Contraindications for sutureless anastomoses included an inner vessel diameter <5 mm or ≥12 mm, a landing zone <2 cm, severe distal stenosis, severe circumferential calcification, or occlusion of the target vessel. Additionally, potential occlusion of a proximal branch off the target vessel with the stent (eg, replaced right hepatic artery originating from the SMA) was a relative contraindication.

### Outcomes

The primary outcomes included immediate technical success, cases of bleeding, occlusion, or thrombosis that required reintervention, and postoperative anastomotic patency at 3 months and 1 year of follow-up with computed tomography angiography (CTA). Other outcomes analyzed included the time to complete the anastomosis, major adverse cardiac events, renal failure requiring hemodialysis, paraparesis, paraplegia, and 30-day mortality.

### Preoperative preparation

All patients underwent preoperative CTA of the chest, abdomen, and pelvis to define the aortic pathology, morphology, and vessel dimensions for stent planning. Special attention was taken to evaluate a target branch's vessel length, diameter at the origin, significant proximal bifurcations, and degree of ostial calcification/stenosis. The branch grafts were made of Dacron (polyethylene terephthalate) and sized to match the target vessel diameter. The Viabahn stent (W.L. Gore & Associates, Flagstaff, AZ) was oversized 10% to 20% to ensure an adequate seal. Thus, a vessel diameter of 6 mm would require a 6-mm Dacron graft limb and an 8-mm by 5-cm Viabahn stent to oversize the graft limb.

All patients were placed on partial or full mechanical circulatory support. The cannulation strategy for cardiopulmonary bypass (CPB) was dependent on the peripheral vascular anatomy and aneurysmal morphology. Repairs of TAAAs were typically done with left heart bypass via the left inferior pulmonary vein and left femoral artery. If it was not safe to cross clamp the aorta proximal to the aneurysm, deep hypothermic (18°C) cardiac arrest with full CPB was used. Repairs of the aortic arch were done with moderate hypothermia (26°C) circulatory arrest and antegrade cerebral perfusion.

### Induction

Patients underwent general anesthesia induction with endotracheal intubation, peripheral intravenous line placement, and invasive arterial pressure monitoring. Simultaneously, on the back table, a Dacron vascular graft was constructed with polypropylene suture and reinforced with surgical adhesive. A four-branched Coselli-style graft was created for TAAAs, and an aortic arch with varying branches was created for arch aneurysms.

### Sutureless visceral debranching during open thoracoabdominal aneurysm repair

For sutureless visceral vessel debranching (Supplementary Video, online only), the patient is placed in the left lateral decubitus position, and a thoracoabdominal incision is made obliquely toward the umbilicus, between the sixth and eighth intercostal space, depending on the extent of the aneurysm. Through this incision, the abdominal contents and chest are accessed. The diaphragm is divided, the origin of the left renal artery is identified, and the kidney is mobilized medially to better facilitate access with the wire and stent and to avoid having the left renal vein obstructing the posterior aorta. After the aorta is dissected and isolated, the patient is placed on CPB and cooled. The aorta is then cross clamped according to the extent of the aneurysm and transected, and the aneurysm is opened longitudinally in preparation for the proximal anastomosis. Both renal artery ostia are identified and cannulated with 9F occlusion catheters and instilled with cold renal perfusate. After the limbs of the graft are oriented toward their respective mesenteric and renal orifices, the proximal aortic anastomosis is performed with running suture. The anastomosis is then de-aired and confirmed to be hemostatic. Next, the distal end of the graft is trimmed to fit the length of the repair, and a 2- to 3-cm longitudinal graftotomy is created with cautery. The graftotomy is created adjacent to the mesenteric vessels and 180° from the right renal artery ostia to facilitate passing the stent. We debranch in a posterior to anterior fashion, starting with the right renal artery, followed by the SMA, celiac artery, and, finally, the left renal artery. The right renal limb of the Dacron graft is trimmed and aligned to the orifice with two braided polyester sutures at the 3- and 9-o'clock positions. The Viabahn stent is marked to ensure only 2 cm of the stent is advanced into the vessel. Ideally, we position the stent 2 cm into the native vessel, with 2 cm as a seal zone in the Dacron graft and 0.5 to 1 cm protruding into the neoaorta. The graft is loaded on a 0.035-in. Rosen guidewire (Cook Medical LLC, Bloomington, IN), and the right renal ostia is accessed via the graftotomy. Once the positioning is satisfactory, the two stay sutures are secured with COR-KNOTs (LSI Solutions, Santa Clara, CA) to align the Dacron graft and target vessel. The stent is deployed, and we confirm the anastomosis is patent, sealed, and hemostatic. The stent is then maximally expanded with a commensurate Armada 35 angioplasty balloon (Abbott Laboratories, Chicago, IL). After the right renal graft is completed, the distal anastomosis is completed with a running suture. A clamp is then placed immediately proximal to the distal anastomosis, and we begin inflow from the common femoral arterial line to perfuse the pelvis and lower extremities. After suturing any bleeding lumbar and intercostal arteries, we proceed with debranching the remaining visceral vessels in the same sutureless fashion. After visceral debranching is completed, we close the graftotomy with a running suture. The thoracic aortic cross clamp is removed, thereby restoring flow to the mesenteric and renal vessels, and the patient is rewarmed to a maximum temperature of 34°C. The thoracoabdominal incision is closed in standard fashion.

On postoperative day 3, as long as no clinical concerns exist for bleeding, dual antiplatelet therapy is initiated with aspirin and clopidogrel for 3 months, followed by lifelong aspirin.

### Sutureless supra-aortic trunk debranching during aortic arch repair

The SATs are debranched with the same sutureless technique described in the previous section, although the variation in the number and configuration of the branch limbs is greater. The innominate artery is typically too large to be debranched with the sutureless technique. The largest Viabahn available (13 mm × 5 cm) is too small to oversize the corresponding Dacron graft. Therefore, the innominate artery is reimplanted in the traditional sutured fashion.

### Postoperative follow-up

The patients were monitored for in situ aortic degeneration and stent patency with clinic visits and CTA at 3 months, 6 months, and 1 year and then yearly afterward.

## Results

During the study period, 26 patients underwent either TAAA or aortic arch repair with a sutureless anastomosis. In total, 50 individual aortic vessels were debranched with the sutureless self-expanding stent anastomosis, including 42 visceral vessels and 8 SAT vessels. The median age was 62.5 years (range, 32-79 years), and 73% (n = 19) were men. Nearly one half of the cases (n = 12) were urgent/emergent, and most patients (n = 14) had undergone previous cardiac interventions. The median time of CPB, aortic cross clamping, moderate hypothermic circulatory arrest, and deep hypothermic circulatory arrest were 293 minutes (range, 60-590 minutes), 169 minutes (range, 60-482 minutes), 21 minutes (range, 12-52 minutes), and 23 minutes (range, 19-70 minutes), respectively (Table II).

**Table II:** Surgical characteristics

Variable	Value
Sutureless anastomosis	
Total	50
Innominate artery	0
Left carotid artery	5
Left subclavian artery	3
Celiac artery	11
SMA	13
Left renal artery	7
Right renal artery	11
Median intraoperative PRBCs, U	8
CPB time, minutes (n = 24)	293 (60-590)
Aortic cross-clamp time, minutes (n = 11)	169 (60-482)
Moderate hypothermic circulatory arrest time, minutes (n = 4)	21 (12-52)
Deep hypothermic circulatory arrest time, minutes (n = 7)	23 (19-70)
Time to complete sutureless anastomosis, minutes (n = 24)	192 (111-324)
Complication	
Stroke	2 (7.7)
Myocardial infarction	0 (0)
Renal failure requiring dialysis	4 (15.4)
Renal failure requiring permanent dialysis	2 (7.7)
Early spinal cord ischemia/paraplegia	1 (4)
Reoperation	
For bleeding	2 (7.7)
For bleeding from sutureless anastomosis	0 (0)
Follow-up imaging completed	21 (80)
Anastomotic kink or stenosis	0 (0)
30-Day mortality	4 (15.4)
DIC	3 (75)
CVA and ischemic colitis	1 (25)

*CPB,* Cardiopulmonary bypass; *CVA,* cerebrovascular accident; *DIC,* disseminated intravascular coagulopathy; *PRBCs,* packed red blood cells; *SMA,* superior mesenteric artery.

Data presented as number, median (range), or number (%).

Excluding the innominate artery (n = 6), the sutureless technique was used to debranch 87.7% of all revascularized branches, with the remaining seven anastomoses created in the standard sutured fashion. The reasons to abort the sutureless technique included severe, circumferential calcification (n = 4) and an inadequate landing zone (n = 2).

Immediate technical success was 100%. Of the 50 sutureless anastomoses, only 1 required minor suturing to obtain hemostasis after deploying the stent graft. The time required to complete the anastomosis was available for 13 of the most recent patients, with 24 sutureless anastomoses completed in an average of 3 minutes, 12 seconds (range, 1 minute, 51 seconds to 5 minutes, 24 seconds). The same cohort had seven sutured anastomoses completed in 3 minutes, 26 seconds (range, 1 minute, 28 seconds to 6 minutes, 7 seconds). These included five innominate and two left common carotid arteries with severe circumferential calcification, precluding the sutureless technique.

No patient required reoperation or reintervention for issues related to the sutureless anastomosis. Two patients required reoperation for bleeding unrelated to the sutureless anastomosis. The first patient was transferred from a referring hospital for emergent repair of a ruptured extent IV TAAA. The initial repair was successful, but the patient remained critically ill due to disseminated intravascular coagulopathy (DIC). No surgical bleeding was identified during reoperation. The patient died within 24 hours of the index operation. The second case was elective repair of an extent II TAAA in a patient with a Marfan syndrome disorder who required revision of the distal aortic suture line on postoperative day 9.

The incidence of stroke, renal failure requiring hemodialysis, bowel ischemia, and spinal cord infarction was 8% (n = 2), 15% (n = 4), 4% (n = 1), and 4% (n = 1), respectively. Perioperative mortality was 15% (n = 4). All the deaths had occurred after thoracoabdominal operations, one half of which were urgent/emergent (Table II). Three deaths were due to DIC and one to cerebral vascular accident and multiorgan system failure. Of those who died of DIC, the first had developed a sudden, large pulmonary embolism and, later, atrial clots and clot extending into the descending aorta. The second had experienced complete hemodynamic collapse after weaning from CPB and developed diffuse bleeding from all the suture lines and raw surfaces. The third patient underwent repair for a ruptured extent IV TAAA and postoperatively experienced intractable hemorrhage from the chest tubes, despite two reoperations that failed to identify a specific source of bleeding.

Postoperative imaging with CTA at 3 months (n = 22), 6 months (n = 21), and 1 year (n = 21) revealed a 100% patency rate of the sutureless anastomoses without any concern for kinking, stenosis, stent migration, or aneurysmal degeneration. No secondary procedures were required to maintain patency of the stents during the follow-up period.

## Discussion

The four-branched graft has become a popular method of aortic reconstruction in patients with TAAAs.^8^^,^^9^ The traditional Crawford inclusion technique, which involves en bloc reimplantation of visceral and intercostal vessels as a Carrel patch into the aortic graft, leaves a considerable amount of diseased aortic tissue prone to patch aneurysm. The prevalence of patch aneurysm development is ∼7.5% after 6 years and can be as high as 18% in patients with connective tissue disorders. This is concerning given that the mortality after reoperation for patch aneurysms is as high as 40%.^3^^,^^4^ Individual branch reconstruction with the four-branch technique obviates this risk by eliminating the patch reconstruction and, instead, creating individual anastomoses between the visceral vessels and branches of the Dacron graft. Separate anastomoses with various graft limb lengths also provides flexibility for reconstruction and helps overcome some of the technical challenges of patch reconstruction when the aneurysm morphology causes long distances between visceral branches.^10^ However, in contrast to a single patch reimplantation, a branched graft increases the number of hand-sewn anastomoses, which are more time consuming to construct and frequently require additional suturing to achieve hemostasis. In complex aortic surgery, any delay to complete an anastomosis prolongs organ ischemia and increases the risk of renal failure, mesenteric ischemia, stroke, spinal cord ischemia, and death.^11^^,^^12^ The risk of neurological complications can arise after only 30 minutes of cross-clamp time; thus, timely repair and reperfusion of organs is paramount.^13^ Another potential problem with the use of the branched graft for TAAA repair is that the visceral vessels, particularly the left renal artery, are at risk of kinking and being placed on tension once the retroperitoneum has been returned to its anatomical position after detorsion at the end of the operation.^5^ One option to mitigate this problem is to intentionally create long graft limbs and wrap them around the main tube graft to ensure an ideal graft/visceral artery configuration after detorsion of the retroperitoneum.

To reduce the number of sutured anastomoses and provide structure to the anastomoses, we deployed a self-expanding stent graft into the visceral or SAT vessels, resulting in creation of an end-to-end, vessel-to-branch graft anastomosis without suturing. To the best of our knowledge, we report the largest experience of using sutureless anastomosis to debranch major aortic vessels during open repair of thoracoabdominal and aortic arch aneurysms.

The sutureless anastomosis proved to be technically reliable, with deployment of the stent graft immediately sealing the anastomosis in all but one case, which was remedied by a single corrective suture. This is advantageous given that traditional debranching commonly requires several corrective sutures to achieve hemostasis in an anticoagulated patient. During short-term follow-up, all the anastomoses were widely patent without evidence of stent graft misalignment. Also, none had required reintervention at ≤1 year postoperatively.

In our experience, the technique helped overcome several cases of difficult anatomy (eg, a very posterior left subclavian artery) that would have otherwise been time consuming to reimplant with arduous suturing during critical aspects of the aortic repair. It also reduced the amount of tissue manipulation and mitigated the risk of narrowing the anastomosis with suture.

Furthermore, the stent graft provides structure and integrity, which could prevent kinking of the revascularized vessel during viscera derotation. Previous experience at our institution has shown the left renal artery anastomosis is particularly vulnerable to twisting or kinking after viscera detorsion and a return to its anatomic position, at times leading to acute kidney injury and chronically, renal artery atrophy (unpublished data).

Although the data regarding the time to complete the sutureless anastomosis was limited and without a true control group, we found the time to complete the anastomosis was reasonable and not longer than that for traditional suturing.

### Complications

The rate of complications such as spinal cord ischemia and renal failure requiring permanent dialysis were similar to the reported outcomes from experienced aortic centers.^11^^,^^14^ Our rates of in-hospital mortality and stroke were slightly higher (15% and 8%, respectively), although our cohort was notably smaller and had more often required urgent/emergent operations.

### Renal failure

Four patients required hemodialysis postoperatively. Only two of these four patients had undergone interventions of the renal arteries. The first patient had had all the visceral vessels debranched with sutureless anastomoses, except for the left renal artery. That patient had presented emergently with a ruptured type B dissection and malperfusion of the mesentery artery, right renal artery, and bilateral lower extremities. The preoperative creatinine was 3.1 mg/dL. Postoperatively, the patient developed diffuse bowel necrosis, multisystem organ failure, and, ultimately, died on the third postoperative day.

The second patient was a 72-year-old woman. She was a current smoker with a history of chronic kidney disease (preoperative creatinine, 2.6 mg/dL), atrial fibrillation, Marfan syndrome, and previous thoracic endovascular aneurysm repair for a type B aortic dissection. She had developed aneurysmal degeneration of her descending aorta. She underwent elective repair of her extent IV TAAA, with all four visceral vessels debranched in sutureless fashion. The aortic arch had not been manipulated, and 18 minutes of deep hypothermic circulatory arrest had been required. On postoperative day 3, she was obtunded, despite weaning from sedation. Imaging revealed acute, multiterritorial (bilateral anterior and posterior circulation) embolic cerebral infarctions and microhemorrhages, believed to have resulted from her atrial fibrillation, given her medical history and lack of aortic arch manipulation. Her residual deficits led to chronic aspiration and the need for a tracheostomy tube and a gastrostomy tube. She had recovered renal function before discharge to a rehabilitation facility.

### Stroke

The only other patient to suffer a stroke was a 60-year-old man with a long history of tobacco smoking. He was incidentally found to have an aneurysm of the aortic root, ascending aorta, and distal arch during lung cancer screening. He developed chest pain while awaiting elective repair and required urgent aortic root and valve replacement, total arch repair, and a frozen elephant trunk.

All the SATs were debranched, but only the left common carotid artery was debranched using sutureless anastomosis owing to the large size of the innominate artery and severe, circumferential calcification of the left subclavian artery. He had required 70 minutes of moderate hypothermic circulatory arrest with antegrade cerebral perfusion. On postoperative day 1, he displayed new-onset left hemiparesis. Imaging demonstrated a small cerebral infarct involving the right subinsular and right frontal lobe in the right middle cerebral artery territory, notably on the contralateral side of the sutureless repair. The etiology was likely a proximal embolic source due to the significant aortic aneurysm repair. His deficits had nearly resolved by the time of his discharge home.

### Literature review

Multiple techniques to create sutureless anastomoses have been described but have largely focused on debranching before hybrid repair,^7^ peripheral revascularization,^15^ or devices that are now discontinued.^16^

Lachat et al^7^ first described telescoping self-expanding stent grafts for renal revascularization during hybrid repairs of abdominal and thoracoabdominal aortic aneurysms. The method, termed VORTEC (Viabahn open revascularization technique) was designed to decrease challenging vessel exposure and anastomotic creation, which would reduce the duration of ischemia and simplify complex aortic repair. Their group has successfully used the VORTEC to debranch a variety of visceral vessels and SATs during hybrid repair of thoracoabdominal and aortic arch pathologies, respectively.^17^, ^18^, ^19^ Overall, they showed their VORTEC method decreased the ischemic times, renal dysfunction rates, and maintained similar patency rates to traditional sutured bypass grafts at mid-term follow-up (≤7 years). However, most reported data on VORTEC has been of hybrid repairs: first, open debranching, followed by endovascular repair of the diseased aorta, either as single- or two-stage procedures. Our technique expands the use of the sutureless anastomosis to one-stage, open repair and maintains the advantages of a four-limb branch graft with the addition of direct visualization of the ostia of all four visceral vessels and avoidance of percutaneous access complications.

Another sutureless anastomosis technology based on the Viabahn endoprosthesis was the Gore hybrid vascular graft (GHVG; W.L. Gore & Associates). The GHVG was a novel expanded polytetrafluoroethylene (ePTFE) vascular prosthesis that included a nitinol-reinforced self-expanding section at the end that allowed for creation of a “sutureless” endovascular anastomosis. Bornak et al^16^ first reported a case of using the GHVG to revascularize the visceral arteries during hybrid repair of a TAAA. Chiesa et al^20^ reported a small cohort of 25 patients receiving renal artery debranching during open TAAA repair with the GHVG. They revascularized 26 renal arteries and demonstrated technical feasibility and short-term patency similar to standard Carrel patch reimplantation. Tsilimparis et al^21^ reported a series of 24 GHVG anastomoses used for visceral debranching in 11 patients with TAAAs. Of the 11 patients, 9 underwent hybrid repair (either two-stage or single-stage) and 2 patients underwent debranching and open TAAA repair.

Although these early studies successfully demonstrated its use in a variety of vessel revascularizations, including a small portion during open TAAA repair, the device had a maximal diameter of 9 mm, limiting its use in many larger vessels. Notably, the product was discontinued by the manufacturer.

Another technique of a sutureless vascular anastomosis, Viabahn Padova sutureless, was described first in a case report of a man with complete superficial femoral artery occlusion and reconstitution at the above-knee popliteal artery.^15^ The technique involved deploying a Viabahn stent into the extensively and circumferentially calcified distal popliteal artery and then suturing an ePTFE graft to the proximal end of the Viabahn. Finally, the proximal end of the ePTFE graft was sutured to the common femoral artery.

### Study limitations

Our study had many limitations. It was a single-center study with one surgeon and a relatively small cohort and was completed within a short period. Also, no control group was included, and the data on the time to complete the anastomosis were limited. Many of these limitations were due to the retrospective nature of the study. The decision to use the sutureless anastomosis technique was at the discretion of the operating surgeon, and most of the cases were TAAAs and not arch pathology (18 vs 8).

This technique is also technically limited by the diameter of the aortic branch. To ensure an adequate seal, the Viabahn diameter must be 10% to 20% larger than the Dacron graft. The largest Viabahn manufactured is 13 mm, which makes debranching larger vessels (ie, the innominate artery) impractical. We have successfully debranched every visceral vessel and SAT, except for the innominate. The stent also requires at least a 2-cm landing zone, but the visceral and SAT branches are relatively large, and we have never encountered this limitation aside from an early branch.

As previously stated, a proximal branch off the target vessel that would be covered by the stent could preclude using this technique. However, this can be easily avoided by evaluating preoperative imaging studies and instead performing a traditional sutured anastomosis. Ultimately, the benefit of quickly completing an otherwise inaccessible, technically arduous anastomosis might outweigh the risk of sacrificing a branch vessel.

Finally, the Viabahn stents are costly. However, we believe that creating a reliably hemostatic anastomosis that is widely patent and resists kinking could justify the cost.

## Conclusions

The sutureless anastomosis technique to debranch the aorta during open aortic aneurysm repair appears to be a safe and effective alternative to traditional sutured anastomosis in select cases. Together, use of the branch graft and sutureless anastomosis, not only avoids the need to incorporate native aorta into the repair, but is also reliably hemostatic, patent, and, in the case of TAAA repair, prevents vessel kinking after visceral derotation. The operative outcomes are acceptable and the follow-up imaging finding encouraging. The next steps include assessing for improvements in perioperative outcomes related to decreased organ ischemia and further evaluating the long-term durability compared with traditional methods of debranching.

## Appendix (online only)

Supplementary Video (online only) Sutureless visceral debranching during open thoracoabdominal aneurysm repair.
